# Care improves self-reported daily functioning of adolescents with emotional and behavioural problems

**DOI:** 10.1007/s00787-021-01812-8

**Published:** 2021-05-30

**Authors:** Vera Verhage, Sijmen. A. Reijneveld, Charlotte Wunderink, Hans Grietens, Josue Almansa, Danielle E. M. C. Jansen

**Affiliations:** 1 Department of Health Sciences, University Medical Center Groningen, University of Groningen, Groningen, The Netherlands; 2 Centre of Expertise Healthy Ageing, Hanze University of Applied Sciences, Groningen, The Netherlands; 3 Lentis Psychiatric Institute Groningen, Groningen, The Netherlands; 4 Faculty of Psychology and Educational Sciences, Parenting and Special Education Research Unit, KU Leuven, Leuven, Belgium; 5 Department of General Practice & Elderly Care Medicine, University Medical Center Groningen, University of Groningen, Groningen, The Netherlands; 6 Department of Sociology and Interuniversity Center for Social Science Theory and Methodology (ICS), University of Groningen, Groningen, The Netherlands

**Keywords:** Adolescence, Mental health, Child development, Longitudinal studies

## Abstract

Emotional and behavioural problems (EBP) have a negative impact on various life domains of adolescents. Receiving care for EBP may improve the functioning of adolescents with EBP, but evidence on long-term outcomes in real-life settings is lacking. We, therefore, investigated self-reported functioning in different life domains of adolescents with various EBP, and the role of care during a three-year period. We used data from the TAKECARE study, which consist of a care and community cohort. We followed adolescents aged 12 and over (*n* = 733) during 3 years over five assessment rounds. Using the Strengths and Difficulties Questionnaire, self-reported functioning was measured in four life domains: home life, friendships, classroom learning and leisure activities. We categorized the respondents into four groups: (1) adolescents without emotional and behavioural problems (*n* = 298); (2) adolescents with emotional problems (*n* = 192); (3) adolescents with behavioural problems (*n* = 80); and (4) adolescents with both emotional and behavioural problems (*n* = 163). The development of functioning over time was analyzed using longitudinal ordinal (probit) regression analyses. Adolescents with both emotional and behavioural problems reported poorer functioning at baseline in all domains and adolescents who received care reported poorer functioning at baseline compared to adolescents who did not receive care. Regarding the change in functioning during the 3 years, adolescents who received care showed improved functioning in all domains. We found improved functioning after care, even if the problems may not have been solved. Psychosocial care can contribute to the functioning of adolescents with EBP, which can have major effects on their future life.

**Supplementary Information:**

The online version contains supplementary material available at 10.1007/s00787-021-01812-8.

## Introduction

Between 10 and 25% of adolescents in the general population suffer from emotional or behavioural problems (EBP) [1, 2]. These problems have a negative impact on various life domains of adolescents, such as education [3, 4], relationships [5] and social functioning [5–7]. Overall, adolescents with EBP experience worse functioning in various life domains compared to their peers without EBP [8, 9]. These difficulties in functioning can persist into adult life [10]. Adolescents with many EBP (e.g. a high SDQ-score) are more likely to meet diagnostic criteria for psychiatric disorders [11, 12]*. *Furthermore, early and accurate detection of EBP in community settings [13], allows for timely intervention and could, therefore, prevents adolescents from being referred to already overburdened child and adolescent mental health care services.

Receiving preventive or specialized care for EBP may improve the functioning of adolescents with EBP in various life domains. For example, studies on the effect of pharmacological treatment for children and adolescents with ADHD found positive effects on the domains of home life (doing family activities) [14], learning at school, and social activities [15]. A study in children and adolescents with obsessive–compulsive disorder found better outcomes after 14 weeks of cognitive behavioural therapy in the domain of home life, but not in the domains of friendships and school [16]. Finally, a literature review reported that care for anxiety disorders showed improved functioning in the domains of school, home life and social [17].

These studies offer support for a positive effect of care on functioning in different life domains, but they have a number of limitations. First, they did not include broad diagnostic groups (i.e., emotional or behavioural problems), but only included specific diagnostic groups (e.g., obsessive–compulsive disorder [16] or ADHD [14]. This limits the generalizability to other EBP. Second, they did not use self-ratings for adolescent functioning but only used parent proxies, thereby missing the personally experienced functioning of adolescents [6, 8]. A third limitation regards the lack of a comparison group of adolescents drawn from the general population. Investigating the role of care solely in a clinical sample may lead to biased results, because adolescents with both EBP and reduced functioning may be more likely to be referred to care compared to adolescents with EBP and no reduced functioning [8]. With these limitations in mind, our aim was to investigate self-reported functioning in different life domains of adolescents with various EBP and the role of care during a three-year period.

## Methods

### Sample and design

We analyzed data from the TAKECARE study, a representative prospective cohort study of children and adolescents in the north-east of the Netherlands who either (1) received care via Preventive Child Healthcare (PCH) (*n* = 309, 23.3%), Child and Adolescent Social Care (CASC) (*n* = 248, 18.0%) or Child and Adolescent Mental Healthcare (CAMH) (*n* = 825, 59.7%) services for emotional and/or behavioural problems (care cohort, *n* = 1382); or (2) were drawn from the general population in the same region (community cohort, *n* = 666). This concerned a stratified random sample of school children, obtained via five primary schools, two secondary schools, and one school for intermediate vocational education, recruited by taking into account the distribution of children across the study region according to age, gender, socioeconomic position, and degree of urbanization. Children and adolescents in both cohorts were eligible for participation if they were aged between 4 and 18 (inclusive), had estimated IQs of 70 and over, and their parent and/or the adolescent was reasonably able to understand Dutch. Participants were included if the parent or the adolescent provided informed consent and completed the baseline questionnaire. The study design was assessed by the local medical ethical committee and approved without the need for a full assessment.

From the north-east of The Netherlands, 3632 participants were potentially eligible. Of these, 632 refused to receive further information about the study by completing the opt-out form that was attached to the written introduction. Of the 3000 remaining eligible participants, 243 were considered to be non-eligible after telephone contact with research assistants because they did not meet the inclusion criteria, resulting in 2757 confirmed eligible participants. Of the confirmed eligible participants, 2048 were included in the study. For the care sample and the community sample, the resulting response rates were 56.6% and 70.3%, respectively [18].

At baseline, differences between respondents and non-respondents in age, gender, rural/urban area and difficulties experienced were small or trivial, with maximum effects sizes of 0.12 [18]. At follow-up, attrition was somewhat higher for children with a non-Dutch background, children who lived in a low-income household or without both biological parents. Respondents and non-respondents did not differ significantly in terms of psychosocial problems [18]. We restricted the current analyses to adolescents aged 12 and over because these completed the adolescent self-report questionnaires (*n* = 733).

### Procedure and measures

Adolescents completed five questionnaires. Baseline measurements were taken from May 2011 to April 2013. The second (T2), third (T3), fourth (T4) and fifth (T5) questionnaires were sent three, 12, 24 and 36 months after the first (T1) questionnaire, respectively. Adolescents were frequently reminded to complete the questionnaire, and after every completed questionnaire they were rewarded with a gift token.

Emotional and behavioural problems (EBP) were measured as internalizing and externalizing problems using the Dutch version of the Strength and Difficulty Questionnaire (SDQ), as reported by the adolescent. The SDQ consists of 25 items describing positive and negative attributes of children that can be allocated to five subscales: emotional problems, conduct problems, hyperactivity/inattention, peer problems and pro-social behaviour [19, 20]. We measured emotional problems as the sum of 10 items related to emotional and peer problems (Cronbach’s *a *adolescents = 0.74 (T1)) and behavioural problems as the sum of 10 items related to conduct problems and hyperactivity (Cronbach’s *a* adolescents = 0.74 (T1)). We categorized the respondents into four groups: (1) adolescents without emotional and behavioural problems (*n* = 298); (2) adolescents with emotional problems (*n* = 192); (3) adolescents with behavioural problems (*n* = 80); and (4) adolescents with both emotional and behavioural problems (*n* = 163). The thresholds of these categories have been derived from extensive research into the psychometric properties of the SDQ [20, 21] and are similar to those in previous research that used the one-year follow-up data of TakeCare [22].

Functioning was measured with the impact supplement of the SDQ: “Do the difficulties interfere with your everyday life in the following areas [home life, friendships, classroom learning and leisure activities]?”, as reported by the adolescents. We reversed the original categories so that a higher score meant better functioning: (1) “a great deal”, (2) “quite a lot”, (3) “only a little” and (4) “not at all”.

Care (yes/no) was measured in the community cohort at T1 with the Questionnaire Intensive Care for Youth (QUINCY) [23, 24], as reported by the parent/caregiver. The question was as follows: “Did you, or your child, have contact with [list of professionals] in the last 6 months because of the emotional or behavioural problems of your child?” Of the adolescents in the community cohort, 30% (84/280) received care. By definition, adolescents in the care cohort received care from preventive child healthcare, child and adolescent social care, and/or child and adolescent mental healthcare services.

Potential confounders regarded age [25, 26] and gender [27, 28]. We also included family composition [29], which was assessed by asking the parent with whom the adolescent lived most of the time. This was categorized into “biological two-parent family” and “other” (e.g., living with one parent, a foster family or a blended/stepfamily). Educational level of the mother (or caregiver, in case the adolescent lived in a foster family) regarded as the highest educational level achieved by the mother or caregiver. All potential confounders were measured during the first measurement round (T1: entry into care).

### Data analyses

First, we described the baseline characteristics per group, i.e. adolescents with only emotional problems, with only behavioural problems, with a combination of EBP and without EBP (emotional, behavioural, both, or none). Second, we compared self-reported functioning per domain (home life, friendships, classroom learning and leisure activities) between these groups during the three-year period and explored how self-reported functioning developed over time. Third, we investigated if care affected the association between groups and self-reported functioning in four life domains. Given that functioning was measured with ordinal four-category variables, the development of functioning over time was analyzed by longitudinal ordinal (probit) regression analyses, with random intercepts and slope predicted by care and group (emotional, behavioural, both or none), and adjusted by gender, age, educational level of the mother and family composition (both biological parents or other). A probit model assumes that there is an underlying standard normal distribution behind the ordinal outcome variable. Regression parameters indicate changes in this underlying normal score, and thresholds determine the cut-off points of the underlying normal distributions that reproduce the proportions for each outcome category. We tested if care modified the effect of EBP at baseline on functioning, with an interaction between EBP and care. Time was included as continuous in years, with value 0 for baseline, and 0.25, 1, 2 and 3 for the subsequent assessment waves. The longitudinal analyses were performed in Mplus 8.4 [30].

## Results

### Baseline characteristics

Table 1 shows the baseline characteristics per group. At baseline, 62% of the sample was aged between 12 and 15; 57% were female. Although 41% (*n* = 298) of the adolescents had no EBP, 57% of this group received care for EBP. Of the adolescents with emotional or behavioural problems, around 80% received care. Of those with a combination of emotional and behavioural problems, 92% received care*.* Adolescents with emotional problems or both emotional and behavioural problems reported poorer functioning in all four domains compared to adolescents with no problems. Adolescents with behavioural problems reported poorer functioning at baseline in home life and classroom learning compared to adolescents with no problems, but they experienced better functioning in leisure activities and friendships, even compared to adolescents with no problems*.*

**Table 1:** Baseline characteristics of adolescents per group regarding emotional and behavioural problems (EBP)

	No EBP (*n* = 298)	Emotional problems (*n* = 192)	Behavioural problems (*n* = 80)	Emotional and behavioural problems (*n* = 163)	Total (*n* = 733)
Adolescent characteristics *n* (%)					
Age					
12–15	183 (61.4)	109 (56.8)	59 (73.8)	102 (62.6)	453 (61.8)
16–18	115 (38.6)	83 (43.2)	21 (26.3)	61 (37.4)	280 (38.2)
Female (vs. male)	147 (49.3)	135 (70.3)	30 (37.5)	105 (64.4)	417 (56.9)
Received care in the past 6 months	171 (57.4)	153 (79.7)	63 (78.8)	150 (92.0)	537 (73.3)
Parent and family characteristics *n* (%)					
Low educational level of the mother	61 (20.5)	53 (27.6)	26 (32.5)	41 (25.2)	181 (24.7)
Family with two biological parents	190 (63.8)	106 (55.2)	47 (58.8)	78 (47.9)	421 (57.4)
Difficulties in functioning *n* (%)^a^					
Home life	55 (18.5)	110 (57.6)	43 (54.4)	121 (74.2)	329 (44.9)
Friendships	37 (12.4)	95 (49.5)	16 (20.3)	109 (66.9)	257 (35.1)
Classroom learning	64 (21.5)	118 (61.5)	49 (61.3)	133 (82.6)	364 (49.7)
Leisure activities	31 (10.4)	90 (47.4)	18 (22.8)	98 (60.1)	237 (32.3)

^a^We dichotomized the variable self-reported difficulties in functioning into “no problems” [not at all] and “problems” [only a little, quite a lot, a great deal], the numbers and percentages of this last category are presented

### Self-reported functioning in four life domains per group over time

**Fig. 1:**
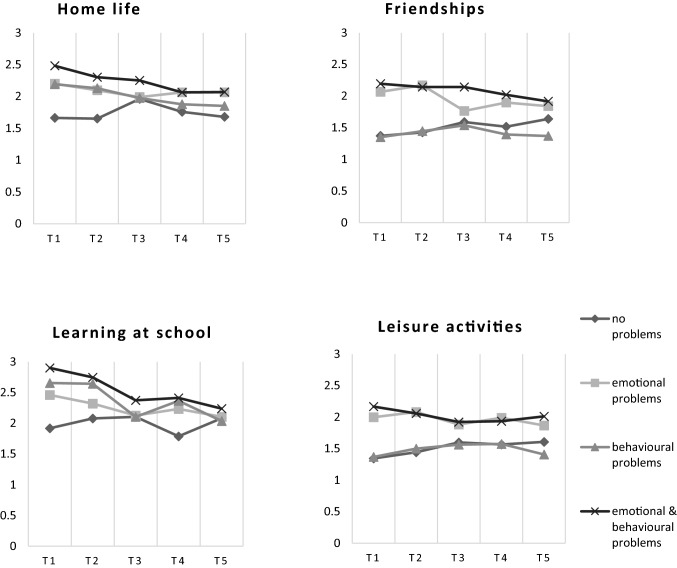
Self-reported functioning (mean) per problem category during a three-year period

Figure 1 shows self-reported functioning per life domain by the group during a three-year period. We found significant differences in self-reported functioning between the four groups in home life, friendships, classroom learning and leisure activities, both at baseline and 3 years later. In Supplemental file 1, self-reported functioning per domain is presented per group at 3 months (T2), 1 year (T3), 2 years (T4) and 3 years (T5) after baseline measurement (T1). After 3 years (T5), adolescents with both emotional and behavioural problems at baseline still reported the poorest functioning, followed by adolescents with emotional and with behavioural problems, respectively.

### The influence of care on self-reported functioning on four life domains over time

Table 2 shows the results of the longitudinal ordinal (probit) regression analysis. Adolescents who received care reported poorer baseline functioning in all four domains compared to adolescents who did not receive care. Girls reported poorer functioning compared to boys in the domains of friendships and home life, and older adolescents showed poorer functioning in classroom learning and leisure activities. We tested if care modified the association between group and functioning with an interaction term. This was not significant (in either intercept or slope), meaning that care does not modify the association between group and self-reported functioning.

**Table 2:** Longitudinal ordinal (probit) regression analysis: self-reported functioning predicted by care and group (emotional, behavioural, both or none), adjusted for age (centered at 12), gender, educational level of the mother and family composition

	Home life	Classroom learning	Friendships	Leisure activities
	Estimate (SE)	Estimate (SE)	Estimate (SE)	Estimate (SE)
Baseline values (intercept)				
Care (yes vs. no)	− 1.57 (0.28)**	− 1.18 (0.24)**	− 1.04 (0.30)**	− 0.91 (0.30)**
Group (ref. = no problems)				
Emotional	− 0.80 (0.26)**	− 1.06 (0.25)**	− 2.06 (0.32)**	− 1.88 (0.30)**
Behavioural	− 1.23 (0.33)**	− 1.57 (0.32)**	0.04 (0.41)	− 0.10 (0.38)
Both emotional and behavioural	− 1.38 (0.27)**	− 1.96 (0.25)**	− 2.35 (0.30)**	− 2.01 (0.28)**
Age	− 0.11 (0.06)	− 0.16 (0.05)**	− 0.13 (0.07)	− 0.19 (0.06)**
Female (vs. male)	− 0.80 (0.20)**	0.13 (0.19)	− 0.52 (0.23)*	− 0.12 (0.22)
Low educational level of the mother (ref. = medium or high)	− 0.24 (0.21)	− 0.02 (0.20)	− 0.00 (0.25)	− 0.22 (0.23)
Other family composition (ref. = both biological parents)	− 0.18 (0.20)	− 0.01 (0.18)	0.26 (0.23)	0.31 (0.21)
Change over time per year (slope)				
Care (yes vs. no)	0.50 (0.13)**	0.48 (0.14)**	0.28 (0.15)*	0.40 (0.16)*
Group (ref. = no problems)				
Emotional	0.13 (0.14)	0.31 (0.13)*	0.58 (0.15)**	0.41 (0.14)**
Behavioural	0.22 (0.20)	0.59 (0.19)**	0.17 (0.24)	0.09 (0.21)
Both emotional and behavioural	0.28 (0.13)*	0.49 (0.13)**	0.51 (0.14)**	0.39 (0.14)*
Age	0.02 (0.03)	0.07 (0.03)*	0.07 (0.03)*	0.02 (0.03)
Female (vs. male)	0.05 (0.11)	− 0.17 (0.11)	0.09 (0.12)	− 0.08 (0.11)
Low educational level of the mother (ref. = medium or high)	− 0.15 (0.12)	0.04 (0.11)	0.03 (0.13)	0.19 (0.12)
Other family composition (ref. = both biological parents)	− 0.11 (0.10)	− 0.12 (0.10)	− 0.10 (0.11)	− 0.06 (0.11)
Mean	− 0.44 (0.18)	− 0.50 (0.17)**	− 0.73 (0.20)**	− 0.65 (0.20)**
Random effects				
Intercept variance	1.82 (0.40)	1.41 (0.32)	2.70 (0.54)	2.06 (0.45)
Slope variance	0.04 (0.09)	0.15 (0.09)	0.18 (0.11)	0.16 (0.10)
Intercept-slope correlation	− 0.26 (0.33)	− 0.22 (0.22)	− 0.36 (0.16)*	− 0.45 (0.17)*
Thresholds***				
1	− 6.32 (0.44)	− 4.63 (0.36)	− 6.81 (0.49)	− 6.33 (0.48)
2	− 4.13 (0.40)	− 2.68 (0.33)	− 4.84 (0.46)	− 4.49 (0.44)
3	− 1.86 (0.37)	− 0.86 (0.32)	− 2.59 (0.43)	− 2.59 (0.42)

^*^Significant differences, *p* < *0.05*

** Significant differences, *p* < *0.01*

^***^ Given that the outcome variables were ordinal and grouped in four categories (a great deal, quite a lot, only a little and not at all), the models included three thresholds, i.e., values at which a respondent migrated to the next outcome level

Regarding changes over time, adolescents who received care reported better functioning compared to those without care in all four domains. Per group, we found that, compared to adolescents with no problems, adolescents with emotional problems improved in functioning regarding friendships and leisure activities, adolescents with behavioural problems improved in functioning regarding classroom learning and adolescents with both emotional and behavioural problems improved in functioning regarding friendships and classroom learning. However, although statistically significant, the estimated differences were quite small. Supplemental file 2 shows an overview of the observed values of adolescents in care and not in care and how they compare over time. This for example, shows that the proportions of adolescents who reported that they experienced problems “only a little” and “not at all” decreased over time for the group not in care and increased for the group in care, especially for “home life” and “classroom learning”. However, these changes were mostly smaller than 0.15. Supplemental file 2 shows that adolescents in the care cohort showed poorer functioning at baseline, but that the community and care cohort showed quite similar functioning after 3 years.

## Discussion

We investigated self-reported functioning of adolescents during a three-year period and the role of care in changes of this functioning. We found that adolescents with both emotional and behavioural problems reported poorer functioning in all four domains (home life, friendships, classroom learning and leisure activities) at baseline, followed by adolescents with only behavioural problems or only emotional problems. We further found functioning to differ per group regarding behavioural problems, with poorer functioning at baseline in home life and classroom learning, but this group of adolescents reported better functioning in leisure activities and friendships. Adolescents who received care reported poorer functioning at baseline in all four domains compared to adolescents who did not receive care. Regarding the change in functioning during the 3 years, adolescents who received care showed an improvement in functioning in all four domains. However, although significant, the estimated improvements were quite small.

This is the first study that investigated the role of a broad variety of psychosocial care on self-reported functioning of adolescents in a real-life setting. We found an improvement in the functioning of adolescents who received care. Previous research in real-life settings (i.e. care as usual) only focusing on problem reduction as an outcome found little benefit of treatment for EBP [31]. Our study implies that the self-reported functioning of adolescents can be improved despite the (chronic) presence of EBP: adolescents who received care might become more successful at handling their problems after receiving care, even if these were not yet solved.

We found that self-reported functioning differed per group regarding behavioural problems, with rates of poor functioning being consistently highest for adolescents with both emotional and behavioural problems. After 3 years, this group of adolescents still reported the poorest functioning. This finding is in line with a large cross-sectional study on quality of life based on functioning in different life domains, showing that children with both emotional and behavioural problems had the lowest self-reported quality of life [32]. It also indicates that co-morbid emotional and behavioural problems lead to worse self-reported functioning in adolescents. These results indicate that EBP impedes functioning and that functioning may be influenced more by the severity than by the type of problem (i.e., emotional or behavioural) [33].

Adolescents who received care reported poorer functioning at the entry of care. At the same time, their functioning in all four domains improved more over time than the functioning of adolescents who did not receive care. The first finding can be interpreted as evidence of care being provided to the adolescents who are in most need. The second finding suggests that receiving care has a positive effect on self-reported functioning of adolescents in different life domains. This could be due to a regression to the mean i.e., that low and high scoring on any scale is due to a random error leading to scores closer to the mean for the second or following measurements [34]. However, it could also indicate the real effects of care. Previous research conducted in clinical settings found some support for the latter explanation of the positive effects of care on functioning in different life (e.g., [14, 16]). Our findings, obtained in a community-based real-life setting, suggest that this positive effect of psychosocial care on the self-reported functioning of adolescents holds true for this broader range of care as well.

We did not find an interaction effect for care and group (no EBP, emotional, behavioural or both emotional and behavioural problems). This may be due to the fact that, in this study, most of the adolescents in care had problems, making that the group without EBP but receiving care was rather small. This may have led to a lack of power to detect an interaction effect for care. Future research should therefore include still larger sample sizes.

Finally, in our study, 57% of the adolescents received care, although they did not report increased levels of EBP themselves. On top of that, we found that adolescents with behavioural problems experienced better functioning in leisure activities and friendships, even compared to adolescents with no problems. One explanation is that adolescents with behavioural problems are perceived as more popular by their peers [35], and therefore experience less difficulties in leisure activities and in friendships. Another explanation may be that adolescents themselves do not consider their problems as serious as parents or health professionals do. Previous research has shown that adolescents report less impact of EBP compared to their parents [36], especially in the case of behavioural problems [37]. This could be due to behavioural problems having a greater impact on family members than on the adolescents themselves [38] and to adolescents failing to attribute problems to themselves [39, 40].

### Strengths and limitations

The strengths of this study are that we investigated outcomes of care regarding functioning instead of problem reduction and that we tackled three limitations of previous research by (a) including broad diagnostic groups (i.e., emotional and behavioural problems); (b) including all types of care provided to adolescents in case of EBP; and (c) using self-reports of adolescents. Including broad diagnostic groups and all types of care enhanced the generalizability of the study [41]. The use of self-reports enabled us to account for the adolescents’ own perception of functioning, which is increasingly recognized as an essential complement of clinical symptomatology and functional impairment in children’s and adolescents’ psychosocial health [6, 42]. Furthermore, this study had a long follow-up (3 years) and a high retention.

The study also has some limitations that are important to acknowledge. First, its observational design limited the potential for inferences on the effectiveness of care. Second, while the response rate at inclusion was relatively low for the care cohort, differences by response status were small, limiting the likelihood of selection bias [18]. Third, the small sample size (due to the categorization in four groups) may have caused an inability to detect an interaction effect for care.

### Implications

The results of this study indicate that adolescents who reported poorer functioning at baseline were more likely to receive care. We also found that adolescents who received care improved in functioning during a three-year follow-up. This implies that practitioners should strive to enhance functioning despite the presence of problems. Cultivating personal strengths and resources may help preserve acceptable levels of well-being despite the presence of symptoms of depression, anxiety, and stress [43]. It could be worth paying attention to this in the training of professionals.

We also found that care provided by various types of psychosocial care providers could improve adolescent functioning. Improved functioning is thus a feasible target for all practitioners in the field of adolescent psychosocial care, making it relevant to learn ways to improve or maintain the functioning of adolescents. This may be reached by reinforcing patient-centred communication with adjustment of the type of treatment to what adolescents themselves consider to be important. Patient-centred communication has been shown to be key to better treatment outcomes (i.e., problem reduction) in psychosocial care for adolescents [44]. This may hold even more true for self-reported functioning.

Future research should include large enough samples to enable the detection of the effect of care on adolescents in separate diagnostic categories. This should provide an insight into how emotional and/or behavioural problems are associated with functioning in different life domains. For such studies, smart recruitment and retention strategies should be used to maximize the validity of research into this hard-to-reach target group. This could help to further substantiate the promising findings of this study regarding the functioning of adolescents with EBP.

## Supplementary Information

Below is the link to the electronic supplementary material.

Supplementary file1 (DOCX 15 KB)

Supplementary file2 (DOCX 16 KB)
