# Supplementary material for: Care improves self-reported daily functioning of adolescents with emotional and behavioural problems
Source: Eur Child Adolesc Psychiatry. 2021 May 30;31(11):1685–93. doi: 10.1007/s00787-021-01812-8 (PMC9666343; doi:10.1007/s00787-021-01812-8)
Supplement: Supplementary file 1 — Supplementary file1 (DOCX 15 KB) [file 787_2021_1812_MOESM1_ESM.docx]

***Supplemental file 1: Self-reported functioning* per domain per group measured: numbers (percentages) with problems, three months (T2), one year (T3), two years (T4) and three years (T5) after T1***

|  | **Home Life** | **Classroom learning** | **Friendships** | **Leisure activities** |
| --- | --- | --- | --- | --- |
| **T2 (n= 667)** |  |  |  |  |
| No problems | 43 (15.8) | 58 (21.2) | 30 (11.0) | 32 (11.8) |
| Emotional problems | 83 (46.6) | 94 (53.1) | 85 (48.0) | 77 (43.5) |
| Behavioural problems | 33 (46.5) | 38 (54.3) | 14 (20.0) | 15 (21.1) |
| Emotional and behavioural problems | 93 (64.1) | 105 (72.4) | 81 (55.9) | 79 (55.2) |
| **T3 (n=658)** |  |  |  |  |
| No problems | 90 (32.3) | 70 (25.6) | 45 (16.5) | 42 (15.4) |
| Emotional problems | 86 (48.9) | 76 (43.7) | 57 (32.6) | 61 (34.9) |
| Behavioural problems | 31 (66.0) | 25 (37.9) | 12 (18.2) | 14 (21.2) |
| Emotional and behavioural problems | 77 (56.2) | 68 (50.7) | 65 (48.5) | 54 (40.3) |
| **T4 (n= 624)** |  |  |  |  |
| No problems | 56 (21.5) | 54 (20.7) | 39 (14.9) | 42 (16.1) |
| Emotional problems | 75 (44.4) | 76 (45.0) | 61 (36.1) | 65 (38.5) |
| Behavioural problems | 21 (33.9) | 22 (35.5) | 11 (17.7) | 13 (21.0) |
| Emotional and behavioural problems | 60 (45.5) | 73 (55.3) | 62 (47.0) | 54 (40.9) |
| **T5 (n= 590)** |  |  |  |  |
| No problems | 47 (18.7) | 65 (25.7) | 46 (18.2) | 43 (17.0) |
| Emotional problems | 75 (46.6) | 67 (41.9) | 58 (36.0) | 55 (34.2) |
| Behavioural problems | 17 (29.8) | 16 (28.1) | 8 (14.0) | 7 (12.3) |
| Emotional and behavioural problems | 60 (50.4) | 58 (48.7) | 55 (46.2) | 54 (45.4) |

* We dichotomized the variable self-reported *difficulties in functioning*into “no problems” [not at all] and “problems” [only a little, quite a lot, a great deal], the numbers and percentages of this last category are presented.
