# Supplementary material for: Care improves self-reported daily functioning of adolescents with emotional and behavioural problems
Source: Eur Child Adolesc Psychiatry. 2021 May 30;31(11):1685–93. doi: 10.1007/s00787-021-01812-8 (PMC9666343; doi:10.1007/s00787-021-01812-8)
Supplement: Supplementary file 2 — Supplementary file2 (DOCX 16 KB) [file 787_2021_1812_MOESM2_ESM.docx]

**Supplemental file 2: Observed proportions of (ordinal) self-reported functioning outcomes for care and non-care groups at baseline (T1) and three years later (T5)**

|  | Observed frequencies | | | |
| --- | --- | --- | --- | --- |
|  | a great deal | quite a lot | only a little | not at all |
| **Friendships** |  |  |  |  |
| *Baseline* |  |  |  |  |
| Not in care | 0.016 | 0.078 | 0.313 | 0.594 |
| In care | 0.078 | 0.165 | 0.322 | 0.435 |
| *After three years (36 months)* |  |  |  |  |
| Not in care | 0.015 | 0.119 | 0.358 | 0.507 |
| In care | 0.051 | 0.140 | 0.350 | 0.458 |
| **Home life** |  |  |  |  |
| *Baseline* |  |  |  |  |
| Not in care | 0.047 | 0.094 | 0.313 | 0.547 |
| In care | 0.093 | 0.270 | 0.384 | 0.253 |
| *After three years (36 months)* |  |  |  |  |
| Not in care | 0.015 | 0.224 | 0.299 | 0.463 |
| In care | 0.056 | 0.192 | 0.407 | 0.346 |
| **Classroom learning** |  |  |  |  |
| *Baseline* |  |  |  |  |
| Not in care | 0.046 | 0.215 | 0.385 | 0.354 |
| In care | 0.216 | 0.332 | 0.274 | 0.178 |
| *After three years (36 months)* |  |  |  |  |
| Not in care | 0.075 | 0.269 | 0.299 | 0.358 |
| In care | 0.113 | 0.244 | 0.305 | 0.338 |
| **Leisure activities** |  |  |  |  |
| *Baseline* |  |  |  |  |
| Not in care | 0.031 | 0.078 | 0.281 | 0.609 |
| In care | 0.087 | 0.140 | 0.306 | 0.466 |
| *After three years (36 months)* |  |  |  |  |
| Not in care | 0.060 | 0.179 | 0.284 | 0.478 |
| In care | 0.061 | 0.136 | 0.299 | 0.505 |
